# Protective Potential of Maresins in Cardiovascular Diseases

**DOI:** 10.3389/fcvm.2022.923413

**Published:** 2022-07-04

**Authors:** Min Liu, Huixiang He, Lihong Chen

**Affiliations:** Advanced Institute for Medical Sciences, Dalian Medical University, Dalian, China

**Keywords:** Maresins, pro-resolving lipid mediator, inflammation, cardiovascular disease, macrophage

## Abstract

Cardiovascular diseases are the leading causes of global mortality. Growing evidence suggests that unresolved inflammation contributes to the chronicity, progression and morbidity of many cardiovascular diseases, thus emphasizing the urgent need to illuminate the mechanisms controlling inflammation and its resolution, for the sake of new effective therapeutic options. Macrophage mediators in resolving inflammation (Maresins) are a family of specialized pro-resolving lipid mediators (SPMs) derived from the ω-3 fatty acid docosahexaenoic acid (DHA). Studies have indicated that Maresins play critical role in initiating the pro-resolving functions of phagocytes, decreasing the magnitude of the overall inflammatory response, and thereby protecting against inflammation-related disorders. In this review, we summarize the detailed actions and the therapeutic potential of Maresins, with a particular emphasis on Maresin-1 (MaR1), in cardiovascular diseases. We hope this review will lead to new avenues to Maresins-based therapies for inflammation-associated cardiovascular diseases.

## Introduction

Cardiovascular diseases (including atherosclerosis, aortic aneurysm, myocardial infarction, and so on) are the leading causes of death and disability in the world. It is known that inflammation is an important feature of many cardiovascular disorders, whereas acute inflammation upon tissue injury would activate adaptive immunity, repair damaged tissue, and eventually restore homeostasis. However, while acute and self-limited inflammatory reaction is protective, the persistent and uncontrolled inflammation amplifies tissue damage and drives the development of various undesirable results. Hence, understanding the mechanisms that control the resolution of inflammation may provide insight into preventing and treating inflammation-associated cardiovascular diseases (1, 2).

In recent years, researchers have demonstrated that the resolution of inflammation is not a passive return to homeostasis, but rather an active process governed by endogenous chemical mediators namely specialized pro-resolving mediators (SPMs) (3). SPMs are a group of fatty acids, including ω-6 arachidonic acid (AA) derived lipoxins, and ω-3 eicosapentaenoic acid (EPA) and docosahexaenoic acid (DHA) derived Resolvins, Protectins and Maresins, as well as n-3 docosapentaenoic acid (DPA) derived SPMs (4–7). In the midst of known SPMs, Maresins were firstly discovered by Serhan et al. in the year 2009 and had displayed widely protective potential in inflammation resolving, wound healing, tissue regeneration and organ protection (8–10). Here, we review the biosynthesis, receptors, and potential protective effects and mechanisms of Maresins in cardiovascular diseases, which may open a new window for seeking optimized anti-inflammatory therapeutic options and improving cardiovascular disease management strategies.

## Biosynthesis and Receptors of Maresins

Maresins are a series of polyhydroxy and polyunsaturated conjugated double bond molecules synthesized from DHA through a series of enzymatic conversion of ω-3 essential fatty acids. At present, the Maresins family mainly includes Maresin-1 (MaR1), Maresin-2 (MaR2), Maresin conjugate in tissue regeneration (MCTRs), and Maresin-like lipid mediators (MaR-Ls). They are named differently according to the position and conformation of hydroxyl and double bond. As reflected in their nomenclature “Macrophage mediators in resolving inflammation,” macrophages are the source of Maresins in their original identification, while neutrophils, platelets and other cell types have afterwards been reported to be able to produce Maresins as well.

**Figure 1:**
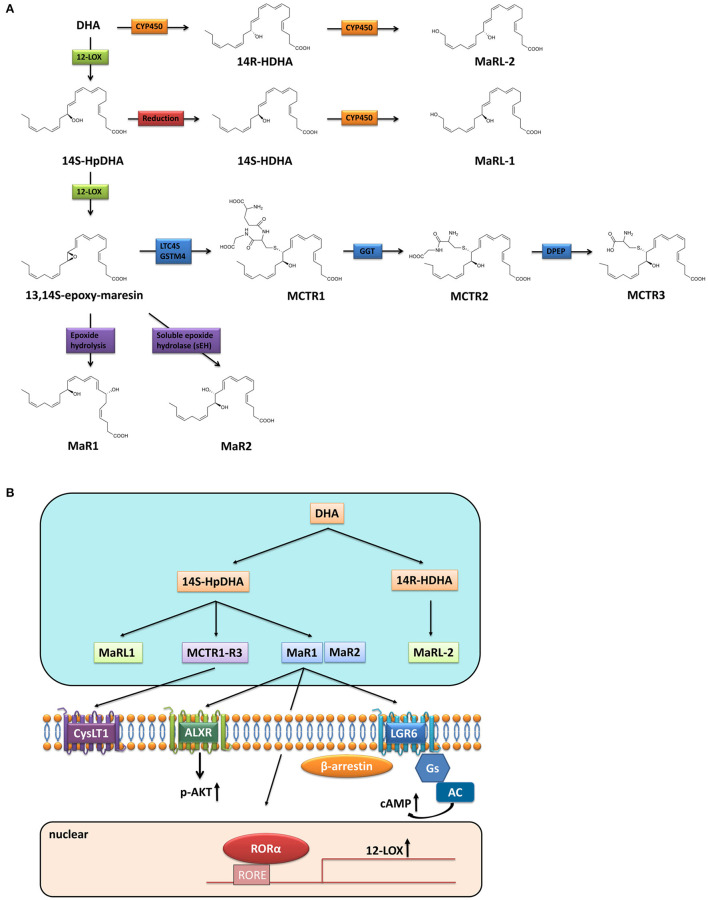
Biosynthesis and receptors of Maresins. **(A)** Biosynthesis of Maresins; **(B)** Receptors of Maresins.

Briefly, after a series of complex desaturation and elongation reactions, ω-3 fatty acids (e.g., a-linolenic acid) is transformed to their higher unsaturated derivatives, EPA and DHA (11). DHA is converted by 12-lipoxygenase (12-LOX) to 14-hydroperoxy-docosahexaenoic acid (14S-HpDHA), and then to the 13,14-epoxide intermediate by the same enzyme denoted 13S,14S-epoxy-Maresin, which is further enzymatically transformed to the bioactive MaRs and MCTRs (12) (Figure 1A). MaR1 is the first member of Maresins family which is produced by enzymatic hydrolysis of 13S,14S-epoxy-Maresin. Whereas, when the epoxide intermediate is followed by conversion via soluble epoxide hydrolase (sEH), it is instantly converted to MaR2 (13). In the biosynthesis of MCTRs, the epoxide intermediate is converted to MCTR1 by leukotriene C4 synthase (LTC4S) or glutathione S-transferase mu 4 (GSTM4) and the conversion of MCTR1 to MCTR2 is catalyzed by γ-glutamyl transferase (GGT). The biosynthesis of MCTR3 is mediated by dipeptidases (DPEP) which cleaved the cysteinyl-glycinyl bond of MCTR2 (14). Different from MaRs and MCTRs, 12-LOX-initiated 14S-hydroxylation or cytochrome P450 (CYP450) catalyzed 14R-hydroxylation and CYP450-initiated ω (22)-hydroxylation are required for MaR-Ls biosynthesis. Leukocytes and platelets convert DHA to 14S-hydroperoxy-DHA (14S-HDHA) via 15-LOX or 12-LOX. 14S-HDHA is further transformed to 14, 22-dihydroxydocosahexaenoic acids (14S, 22-diHDHA) named MaR-L1 through CYP450-catalyzed ω (22)-oxidation. Alternatively, when DHA is transformed to 14R-HDHA by CYP450, 14R-HDHA will be converted to 14R, 22-diHDHA (MaR-L2) (15).

Extensive studies have illustrated that pro-resolving activities of SPMs occur through activation of one or more G protein-coupled receptors (GPCRs) (16, 17). Similarly, after screening hundreds of GPCRs, Serhan et al. found that MaR1 is a ligand for leucine-rich repeat containing G protein-coupled receptor 6 (LGR6), whereas MaR1 stimulates efferocytosis, enhances phagocytosis, and reduces chemotaxis of polymorphonuclear leukocytes and monocytes/macrophages in LGR6-dependent manners via coupling a Gαs protein to stimulate cAMP (4). Besides, studies have shown that MaR1 exerts its function via interacting with lipoxin A4 receptor (ALXR) in several inflammatory disease model, and the salutary effect of MaR1 could be blocked by ALXR antagonist (18, 19), thus ALXR might be another GPCR for MaR1, although the direct binding of MaR1 to ALXR has not been explored. Furthermore, MaR1 is also considered as an endogenous ligand for nuclear receptor retinoic acid-related orphan receptor α (RORα), thus MaR1 enhances the expression and transcriptional activity of RORα and thereby increases the M2 polarity of liver macrophages (20). As a consequence, MaR1 presents cell-type and receptor-dependent actions (Figure 1B). While MCTRs do not directly activate LGR6 (4), they functionally interacts with human cysteinyl-LT receptor (CysLT1) to reduce leukotriene D4 (LTD4) signals (21). Unlike MaR1, the receptor of MaR2 has not been identified yet. There is also no information on the receptors of MaR-Ls and this warrants further investigation.

## MaR1 and Atherosclerosis

Atherosclerosis is a multifocal chronic inflammatory disease of medium sized and large arteries which is the primary cause of fatal cardiovascular diseases (22). Analysis of atherosclerotic lesions in patients with acute myocardial infarction demonstrated that inflammation is the crucial factor leading to plaque rupture and surface plaque erosion (23). A critical aspect is the failure of inflammation resolution, which contains the inhibition of inflammatory cell influx, promotion of inflammatory cell egress, and clearance of apoptotic cells (24). A clear understanding of the process of inflammation and resolution is important to comprehend the complex process of atherosclerosis progression.

Consistent to the concept that an imbalance between specialized pro-resolving and pro-inflammatory lipid mediators promotes instability of atherosclerotic plaques, Viola et al. and Fredma et al. recently reported that there is a significant decrease of pro-resolving MaR1 and/or Resolvin D1/2 (RvD1/2) and increase of the pro-inflammatory leukotriene B4 (LTB4) through the development of early and advanced stages of atherosclerosis in mice (25, 26). By measuring the lesion and necrotic core sizes, macrophage and smooth muscle cell contents, collagen and fibrous cap thickness, Viola et al. demonstrated a significant positive correlation for LTB4 and a negative correlation for MaR1 and RvD2 with vulnerability plaque index. Moreover, therapeutic delivery of RvD1 or a combination of MaR1 and RvD2 both markedly halted atheroprogression and reestablished the balance of SPM to pro-inflammatory leukotrienes. It is well-known that alternatively activated macrophages (M2 macrophages) play critical role in suppressing inflammation and endocytosing cellular debris (24). Dalli et al. have showed that MaR1 has the function of affecting the phenotype of macrophages, tending to polarized toward M2 type, increasing efferocytosis, phagocytic ability, as well as increasing tissue regeneration (6, 27), implying that MaR1 may retard atherogenesis by promoting the inflammation/resolution balance shifted toward resolution. Indeed, by staining M1 and M2 type macrophage specific markers in aortic root sections, an increasing M2 macrophage fraction was shown upon MaR1/RvD2 treatment. Moreover, *in vitro* experiments showed that MaR1 and RvD2 didn't influence the expression of collagen production as well as inflammation genes in smooth muscle cells, while when smooth muscle cells were co-cultured of TNF-activated macrophages, MaR1 and RvD2 increased the mRNA levels of collagen production related genes. These data are consistent with the concept that MaR1 and RvD2 generally act on macrophages, modulating macrophage polarization toward a pro-resolving phenotype, thus suppressing local inflammatory phenotype. This in turn acts on the adjacent smooth muscle cells and stimulates collagen production and stabilizes atherosclerotic plaque (25). Noteworthy, although administration of resolving mediators MaR1 and RvD2 in combination displays promising effects on atheroprogression, given that the possible distinct roles for these two lipid mediators in addition to leukocyte-related functions (28), clarifying the specific role of MaR1 in atherosclerosis is necessarily required.

In addition to the mechanisms mentioned above with restoration of pro-resolving macrophages, *in vitro* experiments demonstrated that MaR1 can also ameliorate LPS-induced pro-inflammatory response, endoplasmic reticulum stress, and cell apoptosis in cultured human umbilical vein endothelial cells (29). Moreover, via up-regulation of cAMP and down-regulation of NF-κB activation, MaR1 significant reduced TNF-α induced monocyte adhesion, reactive oxygen species generation, and pro-inflammatory mediators release in cultured human vascular endothelial and vascular smooth muscle cells (30). These data suggest that MaR1 may also protect against atherosclerotic reactions in vascular endothelial and/or smooth muscle cell-dependent manner. However, the exact role of MaR1 in vascular function during atherogenesis is still not clear.

Increasing evidence has also shown that lymphocytes, particularly T cells, are critical players for the pathogenesis of atherosclerosis. MaR1 indeed has been recognized as an important modulator on adaptive immunity, whereas it suppressed the induction of effector T cells and promoted the induction Treg, thus decreased the production of pro-inflammatory cytokines and increased the production anti-inflammatory cytokines (31). The correlation between MaR1 and Treg/Th17 cell balance has also been investigated in rheumatoid arthritis and other inflammatory diseases (32, 33). However, the direct link between the action of MaR1 on lymphocytes and atherogenesis and other cardiovascular diseases is so far unknown and requires future interpretation. Nevertheless, MaR1 is most likely to be beneficial to atherosclerosis and represents a new window for therapeutic intervention for atherosclerotic diseases.

## MaR1 and Abdominal Aortic Aneurysm

Abdominal aortic aneurysm (AAA) is related to the degradation of the atheromatous aorta elastic media. It is commonly believed that inflammation is associated with AAA development and contributes to elastin breakdown, smooth muscle cell (SMC) phenotypic switching, and extracellular matrix degradation (34, 35). Most recently, Elder et al. reported that MaR1 treatment significantly suppressed murine aneurysm formation in a topical elastase AAA model (36). Mechanistically, MaR1 promotes macrophage-dependent efferocytosis of apoptotic/necrotic SMCs and reduced vascular remodeling by increased TGF-β2 secretion. In SMC-TGFβ2r^−/−^ mice, MaR1-dependent alleviation of AAA formation and increase of efferocytosis of SMCs is abolished. Furthermore, blockade of LGR6 receptor obliterated the protective effects of MaR1 on AAA formation and vascular remodeling (36).

## MaR1 and Other Inflammatory Vascular Diseases

Inflammation is also important pathology in neointimal formation and restenosis after angioplasty. In a mouse model of carotid ligation arterial injury, MaR1 significant reduced early proliferation of VSMCs in the arterial wall and inhibited vascular remodeling through reducing neutrophil and monocyte/macrophage recruitment, as well as increasing M2 polarization in the arteries (37). Upon thrombogenesis, MaR1 has been reported to regulate platelet hemostatic function by enhancing platelet aggregation and spreading, while suppressing release of pro-inflammatory and prothrombotic mediators (38). All these observations point to the therapeutic rationale for MaR1 in inflammatory vascular diseases, modulation of inflammation resolution by MaR1 may provide new directions to limit the vascular injury response and maintain vascular homeostasis.

## MaR1 and Myocardial Infarction and Cardiac Remodeling

Inflammation is critical to the onset and progression of cardiac remodeling and heart failure. While acute inflammatory response usually coincides with active resolving phase, SPMs may act as a guide for resolution of inflammation in the injured myocardium following acute myocardial infarction. Halade et al. illustrated that the abundance of MaR1 and other members of SPMs was significantly enhanced in the infarcted left ventricle within 24 h after myocardial infarction in mice. Moreover, they proved that the splenic macrophages recruited to the infarcted left ventricle represent the major source of these SPMs, highlighting the importance of macrophage-derived SPMs, including MaR1, in retarding heart failure (39). In addition to its dominant anti-inflammatory actions in cardiac macrophages, Wahyuni et al. illustrated that MaR1 can also induce physiological cardiomyocyte hypertrophy through RORα/IGF-1/PI3K/Akt pathway (40). However, the direct action and mechanism of MaR1 on pathological cardiac remodeling and ischemic cardiac injury has not been explored.

## MaR1 and Sepsis-Associated Cardiac Injury

Sepsis is an inflammatory lethal syndrome and about half of patients with sepsis develop cardiac dysfunction. By detecting the peripheral blood lipid mediator profiles in sepsis patients, Dalli et al. recently reported that patients with sepsis had differentially expressed SPMs, this was correlated with survival and clinical outcomes (41). Similarly, in cecal ligation and puncture induced sepsis mouse model, Chen et al. observed significant reduction of multiple SPMs production in the heart tissues, whereas the levels of MaR1 had a ~6-fold reduction (42). Moreover, Li et al. demonstrated that MaR1 treatment remarkedly decreased the serum lactate dehydrogenase and creatine kinase levels and improved LPS-induced cardiac dysfunction in mice via promoting M2 macrophage differentiation, alleviating inflammatory response, and reducing oxidative stress (43). Taken together, administration of MaR1 may be beneficial in reducing sepsis-induced cardiac injury.

## MaR1 and Hypertensive Cardiovascular Disease

Inflammation has been demonstrated as a major component of hypertension and hypertension related organ damage, including peripheral vascular resistance and heart dysfunction. Although the direct relationship between MaR1 and hypertension has not been experimentally tested, by comparing the serum levels of lipoxin and Resolvins in hypertension and normotension patients, Yücel et al. found that the lipoxin A4 (LXA4), RvD1 and Resolvin E1 (RvE1) levels were negatively correlated with systolic blood pressure and total cholesterol levels, implying that reduced SPMs may serve as indicators of the development of hypertension (44). Indeed, RvD1, which is also derived from DHA, had shown protective potential in angiotensin II induced hypertension and cardiac remodeling (45). Resolvins also showed potential to combat pulmonary arterial hypertension and inhibit pulmonary vessel constriction (46, 47). Therefore, it is reasonable for us to expect that MaR1 exhibits beneficial effects on hypertension and hypertensive cardiovascular disease.

## MaR1 and Cardiovascular Metabolic Syndrome

Mounting evidence indicates that patients with metabolic disorders, including obesity, diabetes mellitus, and non-alcoholic fatty liver disease (NAFLD), are at increased risk of cardiovascular events and have a higher cardiovascular morbidity and mortality (48, 49). Metabolic inflammation has been considered as a key process that contributes to multiorgan morbidity (50). Most recently, Fang et al. explored the association between serum MaR1 levels and NAFLD, they found the circulating MaR1 levels were decreased in patients with NAFLD and a negative correlation between NAFLD and serum MaR1 concentrations was identified, indicating that MaR1 might play an important role in the development of NAFLD (51). Similarly, plasma MaR1 concentration was decreased in type 2 diabetic patients and was closely associated with obesity, impaired glucose and lipid metabolism, and enhanced insulin resistance (52). In ob/ob and diet-induced obese mice, MaR1 treatment could inhibit macrophage recruitment, reduce the ratio of M1:M2 macrophages, increase anti-inflammatory adipokine expression, and eventually improve insulin sensitivity and reverse adipose tissue dysfunction (53). MaR1 ameliorated obesity-associated liver steatosis by decreasing lipogenic enzymes, inducing fatty acid oxidation, and activating autophagy through AMPK signaling pathway (54). Moreover, Jung et al. illustrated that MaR1 ameliorated NAFLD via AMPK/SERCA2b-mediated suppression of endoplasmic reticulum stress (55).

Non-alcoholic steatohepatitis (NASH) is a potential progressive liver diseases as a subtype of NAFLD (56). Chronic activation of the hepatic pro-inflammatory immune responses play a vital role in the pathological process of NASH (57). Liver macrophage is a key regulator of hepatic homeostasis by releasing cytokines and modulating immune response in NASH (58). As an endogenous ligand of nuclear receptor RORα, MaR1 was able to increase the expression and transcriptional activity of RORα and thus promoted the M2 polarity of liver macrophages, thus protected mice from high fat diet-induced NASH. More interestingly, activation of RORα can in feedback increase the level of MaR1 through transcriptional induction of 12-LOX, the key enzyme in MaR1 biosynthesis, thus reestablishing the MaR1/RORα/12-LOX autoregulatory circuit may provide a potential therapeutic strategy for treating NASH (20).

## MaR2, MCTRs, And MaR-Ls in Cardiovascular Diseases

MaR2 is identified in Maresins metabolome in macrophages via conversion by human 12-LOX followed by sEH, displayed potent anti-inflammatory and pro-resolving actions (13). In the zymosan injection induced peritonitis mice, MaR2 revealed the potential anti-inflammatory action, although MaR2 is equivalent to MaR1 in restricting neutrophil infiltration, MaR1 is more effective in enhancing macrophage phagocytosis than MaR2 (13). However, the anti-inflammatory effect of MaR2 in cardiovascular diseases has never been confirmed, this warrants further investigation.

Inflammation resolution is an active process which can lead to tissue regeneration. MCTRs were identified as evolutionarily conserved chemical signals that are peptide-lipid conjugated mediators in the Maresins biosynthesis and functionally communicate during resolution of inflammation to activate tissue regeneration (59, 60). With regard to MCTRs and cardiovascular disorders, Yang et al. found that post-treatment with MCTR1 can significantly improve sepsis-induced cardiac dysfunction. Mechanistically, MCTR1 reduced neutrophil chemotaxis and infiltration in LPS-stimulated hearts via attenuating IL-17A production from γδT cells (61). Moreover, Chiang et al. demonstrated that MCTRs can functionally block LTD4-stimulated negative inotropic action on tunicate hearts and antagonize LTD4-initiated vascular leakage (21). Despite these findings, the role of MCTRs in cardiovascular pathogenesis are still largely unknown. Also, we are still far from understanding the role of MaR-Ls in cardiovascular diseases.

## Conclusions and Perspectives

**Figure 2:**
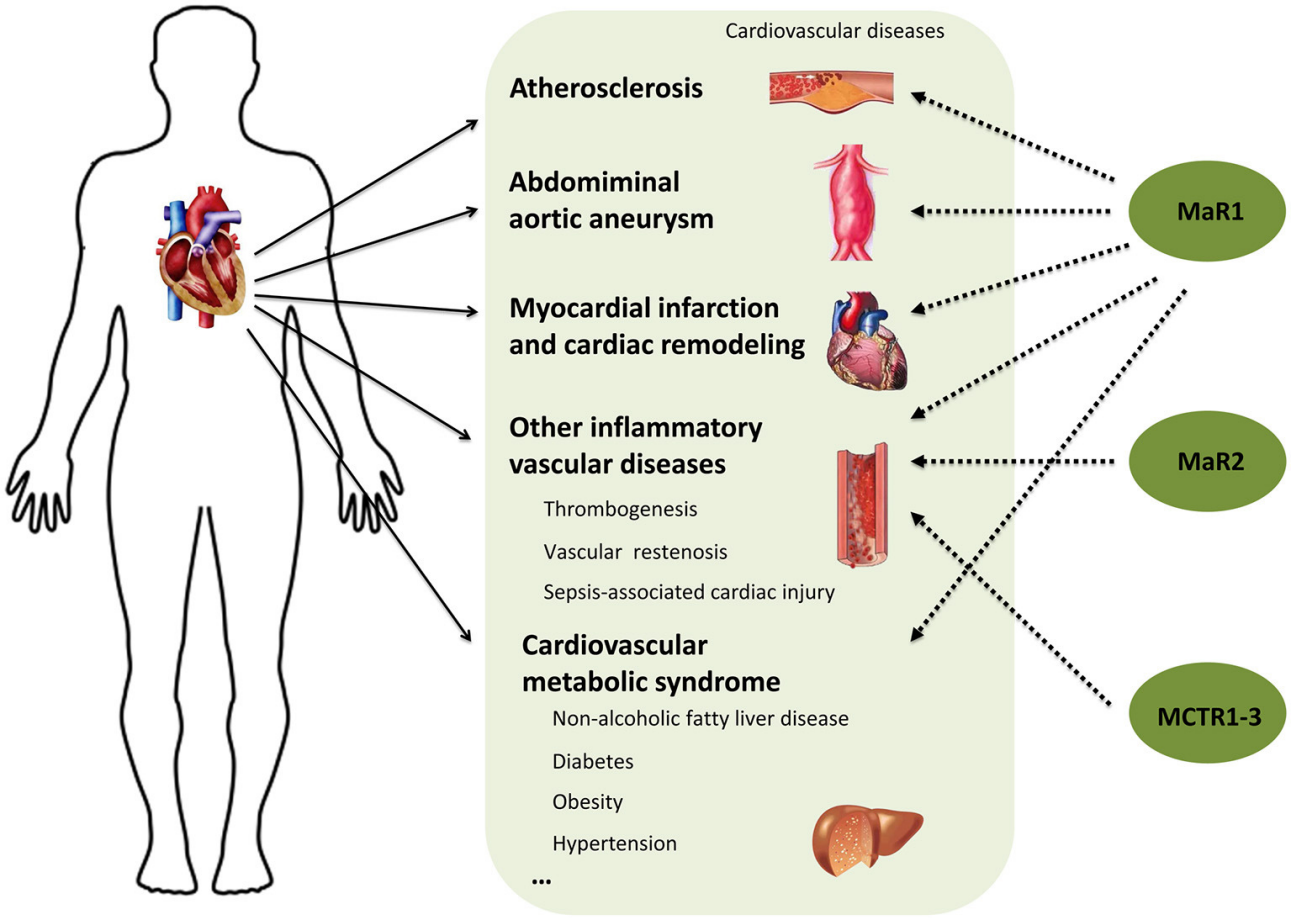
Protective actions of Maresins in cardiovascular diseases.

A large number of studies have shown that inflammation and its resolution are central to most clinically relevant cardiovascular diseases. Although targeting inflammation has been shown to effectively reduce mortality, it has confirmed that anti-inflammatory therapies also enhance the risk of impairing host defense. Thus, boosting inflammation resolution without being overtly immunosuppressive via supplementation of pro-resolving mediators has become new strategy for the treatment of cardiovascular diseases. Maresins are a new family of bioactive lipid mediators synthesized from an ω-3 polyunsaturated fatty acid DHA and have displayed strong anti-inflammatory and pro-resolving abilities and broad beneficial actions in many inflammatory diseases (62, 63). In the current review, by overviewing the studies of Maresins, MaR1 in particular, in the common inflammation associated cardiovascular diseases (Figure 2), we can conclude that administration of MaR1 may serve as a promising therapeutic option and it will be of great significance to synthesize drugs based on endogenous Maresins for the treatment of cardiovascular disease. However, many important questions are still largely unanswered and require in-depth investigation. For example, (1) What is the specific receptor and downstream signaling pathway for MaR1 in a particular cardiovascular disease model? (2) In addition to its anti-inflammatory and pro-resolving activities on monocytes/macrophages and neutrophils, does MaR1 exhibit direct actions on lymphocytes and the mediated immune responses in cardiovascular tissues? (3) What is the effect of MaR1 on cardiomyocytes? fibroblasts? and/or other resident cardiovascular cell types? (4) Do other Maresins exhibit similar protective effects and mechanisms as MaR1 in the anti-inflammatory therapy for cardiovascular diseases? Finally, most of the current evidence are from animal studies, more clinical studies are definitely required to provide better understanding in patients.

## Author Contributions

ML prepared the manuscript draft. HH carefully checked the manuscript and the references. LC strictly commented and amended the paper. All authors contributed to the final version of this review.

## Funding

This work was supported by the National Natural Science Foundation of China (Grant No. 32071157).

## Conflict of Interest

The authors declare that the research was conducted in the absence of any commercial or financial relationships that could be construed as a potential conflict of interest.

## Publisher's Note

All claims expressed in this article are solely those of the authors and do not necessarily represent those of their affiliated organizations, or those of the publisher, the editors and the reviewers. Any product that may be evaluated in this article, or claim that may be made by its manufacturer, is not guaranteed or endorsed by the publisher.
